# Experimental Study on Mechanical and Damage Evolution Characteristics of Coal during True Triaxial Cyclic Loading and Unloading

**DOI:** 10.3390/ma16062384

**Published:** 2023-03-16

**Authors:** Chongyang Jiang, Lianguo Wang, Ke Ding, Shuai Wang, Bo Ren, Jiaxing Guo

**Affiliations:** State Key Laboratory for Geomechanics and Deep Underground Engineering, China University of Mining and Technology, Xuzhou 221116, China

**Keywords:** true triaxial cyclic loading and unloading, mechanical properties of coal, residual strain characteristics, energy dissipation, damage evolution law

## Abstract

Research on the mechanical properties and damage evolution of coal during true triaxial cyclic loading and unloading is of great significance for maintaining the long-term safety and stability of underground engineering structures in coal mines. In this paper, firstly, the deformation, strength and fracturing characteristics of coal during true triaxial loading and true triaxial cyclic loading and unloading were analyzed. Then, the residual strain characteristics, energy distribution and evolution of coal were systematically studied. Additionally, the damage evolution laws of coal during cyclic loading and unloading were quantitatively analyzed from the perspectives of residual strain and energy dissipation, respectively. The damage evolution law based on residual strain showed that when the intermediate principal stress was high, the damage to coal was directional. With the increase in cyclic load, the coal damage variables in the directions of $\sigma_{1}$ and $\sigma_{3}$ increased exponentially, while that in the direction of $\sigma_{2}$ increased quadratically. The damage evolution law based on energy dissipation showed that the coal damage variable increased exponentially with the increase in cyclic load. With the increase in $\sigma_{2}$, the increasing speed of coal damage variable decreased first and then increased. The damage variables established based on residual strain and energy dissipation can both reveal the damage deterioration mechanism of coal during true triaxial cyclic loading and unloading, which is of great theoretical and engineering significance for scientifically evaluating the stability of underground coal and rock engineering and preventing the occurrence of major geological disasters.

## 1. Introduction

In the underground engineering of coal mines, such as roadway tunneling and coal seam mining, coal will be frequently subjected to cyclic loads such as drilling, blasting and mining, and the stress on coal body will change periodically [1,2,3]. During long-term cyclic loading, coal will be gradually damaged, destroyed and even become unstable, thus affecting the stability of engineering structures. In addition, in the underground engineering of coal mines, coal is usually in a three-dimensional unequal stress field ($\sigma_{1}$ > $\sigma_{2}$ > $\sigma_{3}$) [4,5,6], that is, a true triaxial stress state. Further understanding the mechanics and damage fracturing evolution of coal in the real stress field and revealing the damage deterioration mechanism of coal during cyclic loading are of great significance for maintaining the long-term safety and stability of underground engineering structures in coal mines.

At present, many experts and scholars conducted in-depth research on the mechanical properties and damage mechanism of coal and rock mass during uniaxial and triaxial cyclic loading and unloading. Gong et al. [7] carried out multi-stage uniaxial cyclic loading and unloading tests on bituminous coal, described the loading and unloading response ratio of bituminous coal by using the statistical law of acoustic emission ringing count, and obtained the damage evolution characteristics and damage laws during the damage process of bituminous coal. Ding et al. [8] implemented cyclic loading and unloading tests of coal samples under different stress levels, studied the deformation and energy evolution characteristics of coal samples during cyclic loading and unloading, and obtained the damage evolution characteristics of coal based on energy dissipation. Li et al. [9] studied the damage evolution characteristics of shale during triaxial cyclic loading and unloading, and established the plastic strain damage model of confining pressure, damage accumulation coefficient and average damage factor. Meng et al. [10] used the MTS815 rock mechanics test system to carry out triaxial cyclic loading and unloading tests on limestone with different surrounding rocks, and studied the strength, dilatancy deformation and damage mechanical properties of rock during cyclic loading and unloading. In addition, many scholars conducted detailed research on the damage and failure of coal and rock mass during cyclic loading and unloading from the perspectives of plastic deformation [11,12], energy dissipation [13,14] and acoustic emission characteristics [15,16] of coal and rock mass.

In recent years, with the development of true triaxial testing machine [17,18,19,20], some scholars explored rock mechanical properties during true triaxial cyclic loading and unloading [21]. Xiao et al. [22] studied the influence of prior cyclic loading and unloading damage on subsequent unloading behavior of sandstone under true triaxial conditions, and analyzed the influence law of cyclic loading and unloading times on strength and deformation characteristics, energy conversion and failure mode of sandstone. Hu et al. [23,24,25] carried out true triaxial cyclic loading and unloading tests on fractured sandstone samples and analyzed the evolution laws of sandstone strength, deformation and energy. Meanwhile, they defined damage variables based on plastic deformation and energy dissipation and obtained the damage laws of fractured sandstone. Gao et al. [4,26] studied the progressive failure process of marble through true triaxial cyclic loading and unloading tests in two paths, and quantified the evolution of rock damage through the results of irreversible strain, dissipated energy, acoustic emission characteristics and scanning electron microscope. It was observed that the true triaxial cyclic loading and unloading test studies mostly focused on hard rocks such as marble and sandstone, while there is little research on soft coal.

This paper takes soft coal as the research object. First, the variation laws of coal strength, fracturing characteristics, deformation and energy during true triaxial cyclic loading and unloading were investigated. Additionally, the damage evolution law of coal body during cyclic loading and unloading was quantitatively analyzed based on residual strain and energy dissipation. On this basis, the damage deterioration mechanism of coal body under cyclic loading was revealed. The research is of great significance for maintaining the long-term safety and stability of underground engineering structures in coal mines.

## 2. Test Method

### 2.1. Sample Preparation

**Figure 1:**
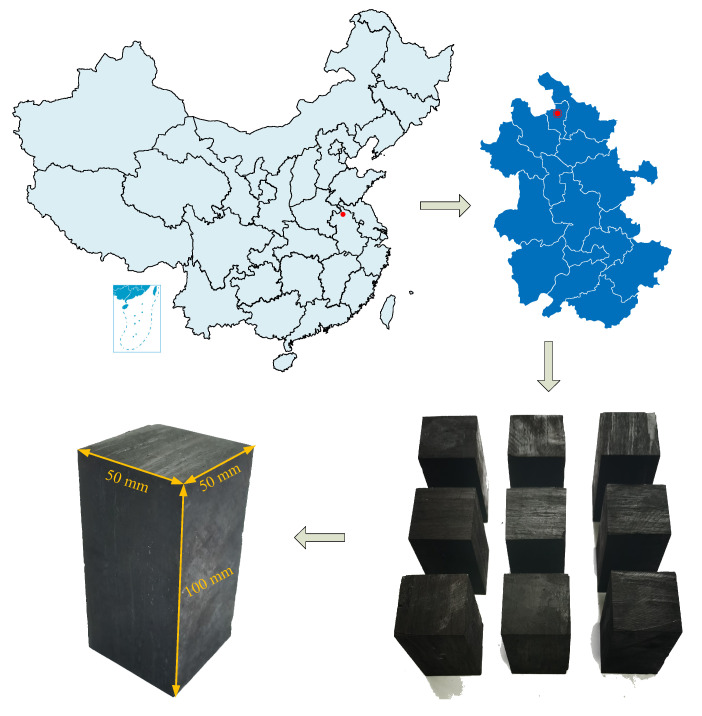
Coal samples of the test.

The coal samples used in this experiment were taken from the Huaibei mining area in China, which was relatively soft with developed primary fractures. According to the requirements of rock mechanics test, the large coal taken from the site was cut and polished to produce standard cuboid samples with the size of 50 mm × 50 mm × 100 mm, whose adjacent faces were perpendicular to each other, and the unevenness of end faces was less than 0.05 mm [17,27,28] (Figure 1). In order to ensure the homogeneity of the samples, the density and ultrasonic wave velocity of the processed samples were tested. Based on the test results, the samples with large discreteness were removed. The average density of the sample was 1.328 g/cm^3^, and the average wave velocity was 1.586 km/s.

### 2.2. Test Equipment

**Figure 2:**
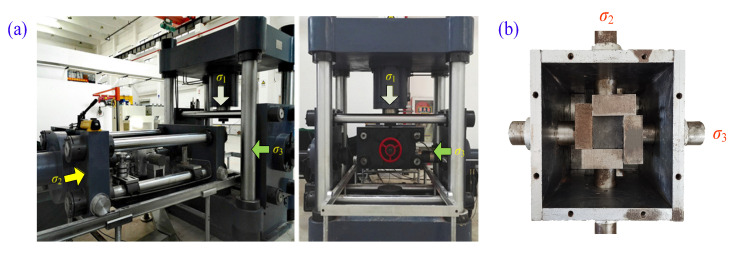
True triaxial test system: (**a**) true triaxial testing machine; (**b**) true triaxial pressure chamber.

This test adopted the true triaxial electro-hydraulic servo loading test system of China University of Mining and Technology. The true triaxial test system is mainly composed of a three-dimensional servo control loading system, a true triaxial pressure chamber and an automatic acquisition system [6] (Figure 2). As seeb in the figure, the three-dimensional servo control loading systems in directions of $\sigma_{1}$, $\sigma_{2}$ and $\sigma_{3}$ were all equipped with independent loading racks, which can realize independent servo loading. The three loading racks were perpendicular to each other in space and together form the loading space of the samples. The three-dimensional servo control loading systems all adopted the rigid loading mode, and the maximum loading capacity was 2000 kN, 500 kN and 300 kN, respectively.

### 2.3. Test Scheme

The tests aimed to study the mechanical properties of coal during true triaxial cyclic loading and unloading and to analyze coal strength, deformation characteristics and damage fracturing behavior under different intermediate principal stresses. Six groups of true triaxial loading tests and true triaxial cyclic loading and unloading tests ($\sigma_{3}$ = 10 MPa, $\sigma_{2}$ = 15, 20, 25, 30, 35 and 40 MPa, respectively) were designed. Considering the discreteness of test results, each group of tests was repeated three times.

#### 2.3.1. True Triaxial Loading Test

**Figure 3:**
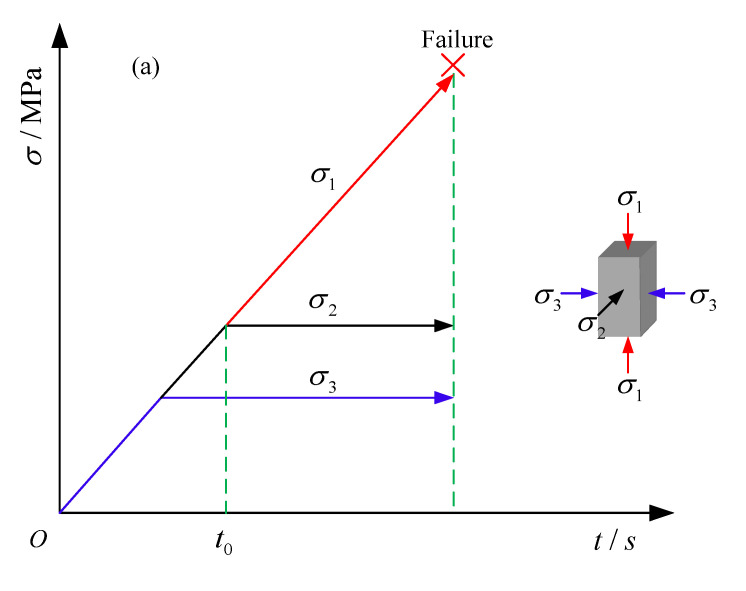

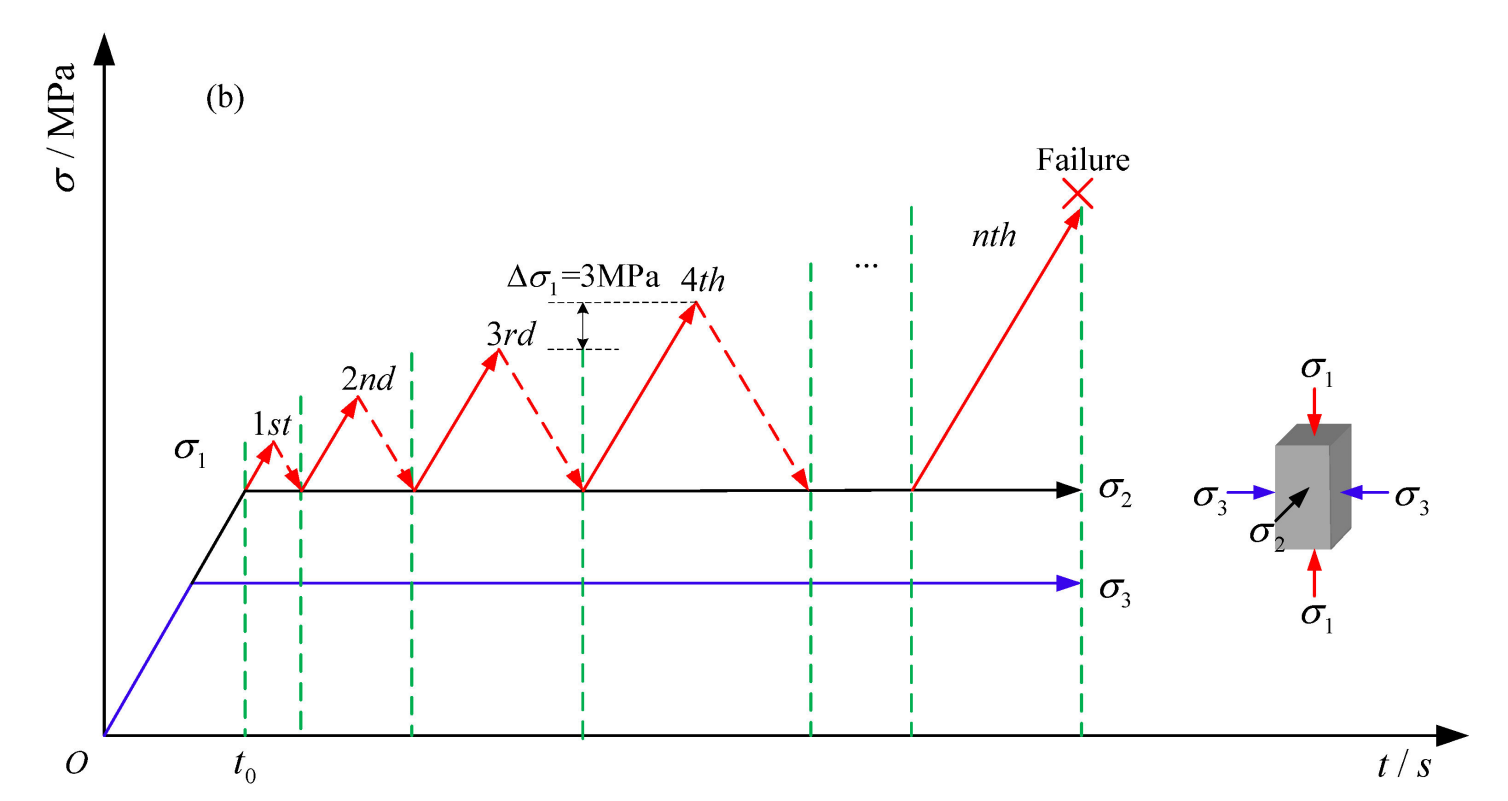
Schematic diagram of test stress path: (**a**) true triaxial loading test; (**b**) true triaxial cyclic loading and unloading test.

In order to obtain the characteristic stress of the coal during true triaxial loading, firstly, the corresponding true triaxial loading test was carried out on the coal. Figure 3a is the loading path of true triaxial loading test. The sample was first loaded to the target hydrostatic pressure state (10 Mpa) in the directions of $\sigma_{1}$, $\sigma_{2}$ and $\sigma_{3}$ at a loading rate of 0.2 Mpa/s by force loading control. Then, $\sigma_{3}$ was kept unchanged, and $\sigma_{1}$ and $\sigma_{2}$ were loaded at a loading rate of 0.2 Mpa/s to the test set value. Finally, $\sigma_{2}$ and $\sigma_{3}$ were kept unchanged, and $\sigma_{1}$ was loaded at a displacement loading rate of 0.002 mm/s until the post-peak residual stage of the sample.

#### 2.3.2. True Triaxial Cyclic Loading and Unloading Test

Figure 3b is the loading path of true triaxial cyclic loading and unloading test. The test adopted stress control cyclic loading and unloading process. On the premise that there were 5–10 cycles before the peak under different confining pressures, the stress gradient of a single cycle was determined to be $\Delta \sigma_{1}$ = 3 MPa by referring to the true triaxial loading test results under different confining pressures. At the beginning of the test, $\sigma_{2}$ and $\sigma_{3}$ were loaded to the target value by the same method as the true triaxial loading test. Additionally, $\sigma_{2}$ and $\sigma_{3}$ were unchanged, and $\sigma_{1}$ was loaded at the loading rate of 0.1 MPa/s. When the axial stress was loaded to the set value, $\sigma_{1}$ was unloaded to $\sigma_{2}$ level by force control at the unloading rate of 0.1 MPa/s. When $\sigma_{1}$ was loaded to the set value of the next cycle, it was unloaded again. The process of loading and unloading was repeated until the post-peak residual stage of the sample was reached.

## 3. Analysis of Test Results

### 3.1. Coal Deformation and Strength Characteristics

Stress–strain curves can effectively describe the whole process of the coal from fracture initiation, fracture propagation to macroscopic fracturing surface appearance, and can effectively reflect the deformation and strength characteristics of the coal. It is an important basis for studying its mechanical characteristics as well as damage and failure characteristics. Therefore, obtaining the stress–strain curve of coal under true triaxial loading and true triaxial cyclic loading and unloading is of great importance for studying the mechanical characteristics and damage characteristics of coal. Because this paper mainly explored the mechanical characteristics of coal under true triaxial conditions, to make the comparative analysis easier, this paper took the deformation of the sample when it was loaded to the initial confining pressure level as the zero point, and only analyzed the data of true triaxial loading and cyclic loading and unloading process.

#### 3.1.1. Deformation and Strength Characteristics of Coal during True Triaxial Loading

**Figure 4:**
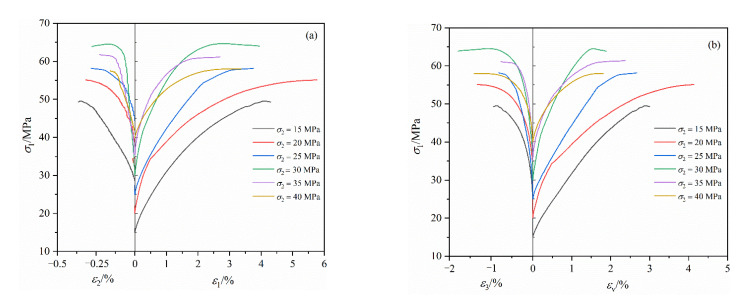
Stress–strain curves of coal during true triaxial loading: (**a**) ($\varepsilon_{1}$,$\varepsilon_{2}$) -$\sigma_{1}$; (**b**) ($\varepsilon_{v}$,$\varepsilon_{3}$) -$\sigma_{1}$.

**Figure 5:**
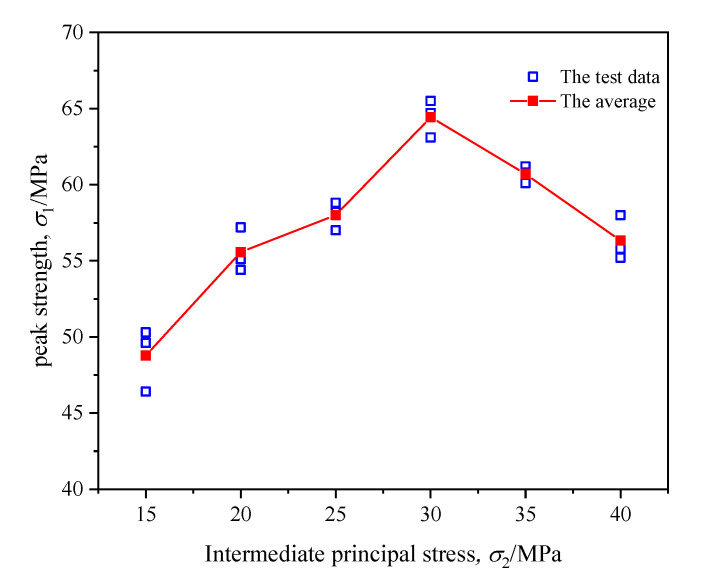
Variation law of coal strength during true triaxial loading.

Figure 4 shows the variation curves of axial stress ($\sigma_{1}$), axial strain ($\varepsilon_{1}$), lateral strain ($\varepsilon_{2}$, $\varepsilon_{3}$) and volume strain ($\varepsilon_{v}$) of coal during true triaxial loading with different intermediate principal stresses. Figure 5 demonstrates the variation curve of coal strength during true triaxial loading.

1. It can be concluded from Figure 4 that the stress–strain characteristics of coal during true triaxial loading were similar under six different stress levels, and the stress–strain relationship of the coal during true triaxial loading mainly included the elastic deformation stage, the fracture propagation stage and the plastic flow stage. The coal sample experienced a short elastic deformation stage before quickly entering the fracture propagation stage. In the fracture propagation stage, the axial stress rose slowly, while the lateral strain increased gradually. Finally, the sample entered the plastic flow stage where the strain of coal increased rapidly; there was no obvious strain softening; and an obvious yield platform appeared. In this stage, macroscopic fractures formed in the coal body, and the plastic flow failure occurred.
2. According to the variation curves of lateral strain ($\varepsilon_{2}$ and $\varepsilon_{3}$) under different intermediate principal stresses in Figure 4, with the continuous increase in $\sigma_{2}$, the slope of the curve of $\varepsilon_{2}$ gradually grew. When $\sigma_{2}$ > 30 MPa, the slope of the curve increased obviously, which showed that large $\sigma_{2}$ will limit the deformation in the direction of $\sigma_{2}$ and drives the sample to deform mainly along the direction of $\sigma_{3}$, thus promoting the destruction. From the curves of volume strain ($\varepsilon_{v}$) under different intermediate principal stresses in Figure 4, it was found that the sample was always in volumetric compression during loading, which indicates that the lateral dilatancy of coal after plastic flow failure was far less than the axial volumetric compression.
3. According to the variation laws of coal strength under different intermediate principal stresses in Figure 5, with the increase in $\sigma_{2}$, the overall strength of the sample showed the trend of first increasing and then decreasing. When $\sigma_{2}$ = 30 MPa, the strength of the sample reached the maximum, and when $\sigma_{2}$ > 30 MPa, $\sigma_{2}$ mainly caused damage to the sample, accelerating the destruction.

#### 3.1.2. Deformation and Strength Characteristics of Coal under True Triaxial Cyclic Loading

**Figure 6:**
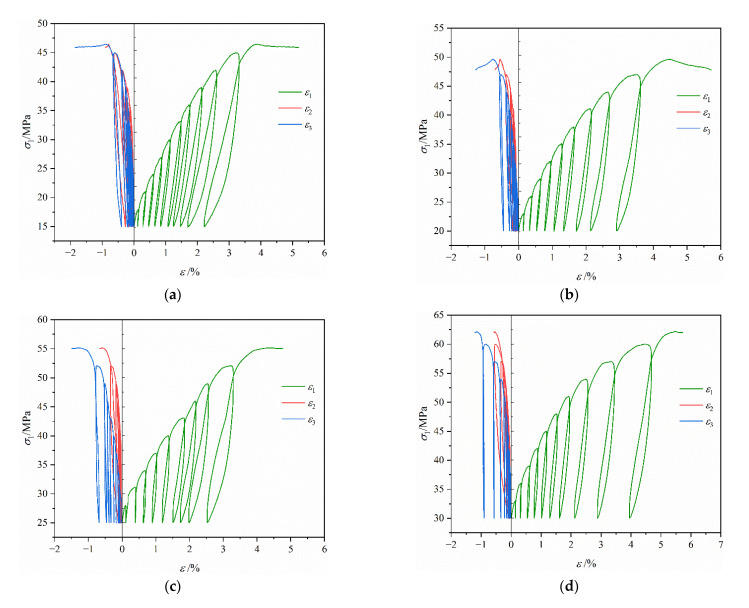

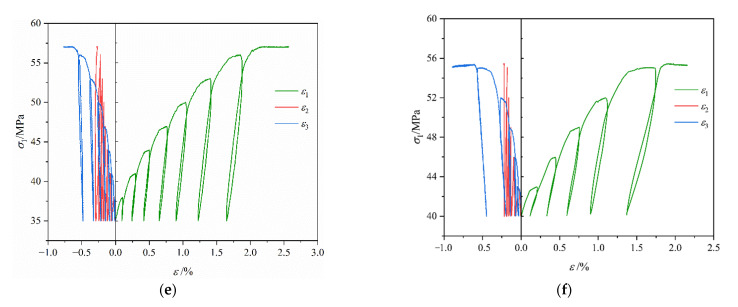
Stress–strain curves of coal during triaxial cyclic loading and unloading: (**a**) $\sigma_{2}$ = 15 MPa; (**b**) $\sigma_{2}$ = 20 MPa; (**c**) $\sigma_{2}$ = 25 MPa; (**d**) $\sigma_{2}$ = 30 MPa; (**e**) $\sigma_{2}$ = 35 MPa; (**f**) $\sigma_{2}$ = 40 MPa.

**Figure 7:**
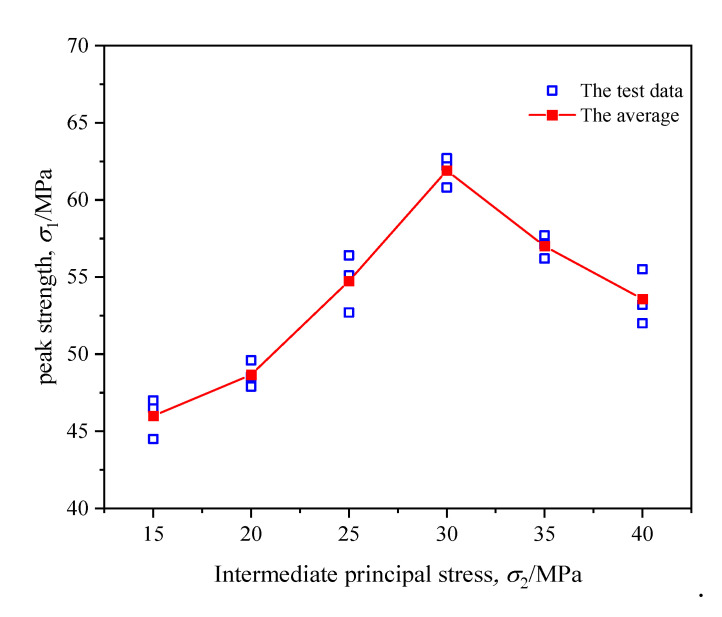
Variation law of coal strength during triaxial cyclic loading and unloading.

Figure 6 reveals the variation curves of axial stress ($\sigma_{1}$), axial strain ($\varepsilon_{1}$), lateral strain ($\varepsilon_{2}$, $\varepsilon_{3}$) of coal during true triaxial cyclic loading with different intermediate principal stresses. Figure 7 demonstrates the variation curve of coal strength during true triaxial cyclic loading. From Figure 6 and Figure 7, the following findings were obtained:

1. In the true triaxial cyclic loading and unloading test, the samples exhibited similar stress–strain characteristics under six different stress levels. In addition, the stress–strain curves of coal had an evident hysteretic effect, forming a hysteresis loop. During each cyclic loading and unloading cycle, the coal body produced certain irreversible deformation in the three directions. As a result, the hysteresis loop kept shifting in the direction of increasing strain. The area of the hysteretic loop in the direction of $\sigma_{1}$ was much larger than those in the directions of $\sigma_{2}$ and $\sigma_{3}$, and the area of the hysteretic loop grew gradually with the increase in the number of cycles, reaching the maximum near the peak strength of coal. Such a result indicates that the internal damage of coal accumulated continuously under the action of cyclic load. Consequently, the fracture expanded continuously, eventually leading to the destruction of the macroscopic fracturing surface.
2. By comparing the stress–strain curves under different intermediate principal stresses, it was found that when $\sigma_{2}$ was 15 MPa or 20 MPa, the difference between $\sigma_{2}$ and $\sigma_{3}$ was relatively small, as was the deformation difference of coal in the directions of $\sigma_{2}$ and $\sigma_{3}$. With the increase in $\sigma_{2}$, the difference between $\sigma_{2}$ and $\sigma_{3}$ enlarged. Accordingly, the deformation difference in the directions of $\sigma_{2}$ and $\sigma_{3}$ also increased gradually. When $\sigma_{2}$ increased to 35 MPa or 40 MPa, the deformation in the direction of $\sigma_{2}$ was restrained, and its lateral deformation was mainly transformed into dilatancy deformation in the direction of $\sigma_{3}$. When plastic failure occurred, deformation in the direction of $\sigma_{2}$ reversed slightly.
3. According to the variation law of coal strength under different intermediate principal stresses, the coal strength during true triaxial cyclic loading and unloading was similar to that during true triaxial loading. With the increase in $\sigma_{2}$, the coal strength rose at first and then fell. When $\sigma_{2}$ = 30 MPa, the coal strength reached its maximum, but the overall strength decreased by 3.9–12.4% compared with that during true triaxial loading.

### 3.2. Characteristics of Coal Fracturing

**Figure 8:**
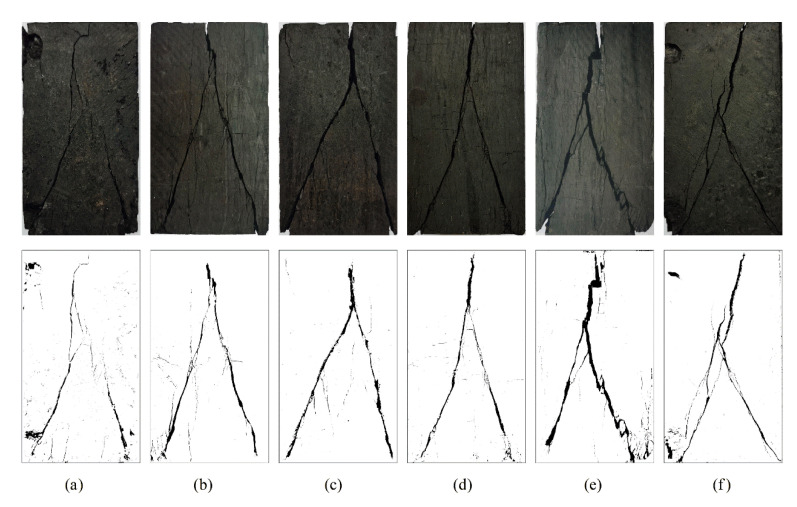
Coal fracturing characteristics during triaxial loading: (**a**) $\sigma_{2}$ = 15 MPa; (**b**) $\sigma_{2}$ = 20 MPa; (**c**) $\sigma_{2}$ = 25 MPa; (**d**) $\sigma_{2}$ = 30 MPa; (**e**) $\sigma_{2}$ = 35 MPa; (**f**) $\sigma_{2}$ = 40 MPa.

**Figure 9:**
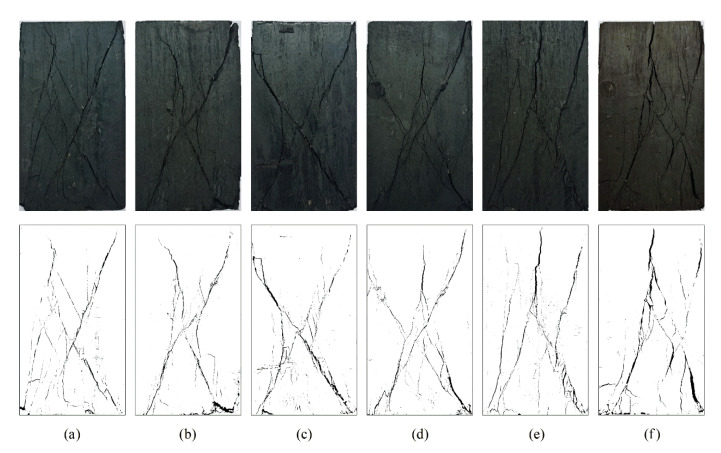
Coal fracturing characteristics during triaxial cyclic loading and unloading: (**a**) $\sigma_{2}$ = 15 MPa; (**b**) $\sigma_{2}$ = 20 MPa; (**c**) $\sigma_{2}$ = 25 MPa; (**d**) $\sigma_{2}$ = 30 MPa; (**e**) $\sigma_{2}$ = 35 MPa; (**f**) $\sigma_{2}$ = 40 MPa.

The macroscopic fracturing characteristics of coal are the final embodiment of its failure process and an important basis for characterizing the deformation and failure mechanism. The states of coal fracturing under the two loading modes are presented in Figure 8 and Figure 9. It was observed that the macroscopic fractures produced by coal fracturing under the two loading paths all appeared in the planes of $\sigma_{1}$–$\sigma_{3}$, primarily because $\sigma_{2}$ > $\sigma_{3}$. $\sigma_{2}$ limits lateral dilatancy in this direction during the loading process, resulting in the occurrence of dilatancy deformation along the direction of $\sigma_{3}$. Finally, macroscopic fractures were formed. The failure modes under the two loading paths were mainly shear failure and tensile failure caused by axial compression. Under true triaxial loading, the sample experienced Y-shaped failure which ran through the whole sample and had few secondary fractures. With the increase in $\sigma_{2}$, the failure mode gradually changed from shear failure to tensile failure. However, the fracture distribution under true triaxial cyclic loading and unloading was more complex than that under true triaxial loading. When $\sigma_{2}$ ≤ 30 MPa, shear failure dominated, which was approximately X-shaped and accompanied by numerous secondary fractures and debris. This phenomenon can be explained as follows: cyclic loading and unloading leads to the accumulation of internal damage in the coal body and the repeated friction of internal structural planes, which causes the change of structural planes and the appearance of more failure planes. Resultantly, the coal body is more broken at the moment of instability and destruction. When $\sigma_{2}$ were 35 MPa or 40 MPa, with the increase in lateral stress difference, $\sigma_{2}$ turned to promote the tensile failure and the main fracture gradually expanded to the vertical direction, finally forming tensile fracture penetrating from the top to the bottom. As a result, the bearing capacity of the coal body weakened.

## 4. Discussion

### 4.1. Evolution Law of Residual Strain and Damage Characteristics

#### 4.1.1. Analysis of Residual Strain Characteristics

**Figure 10:**
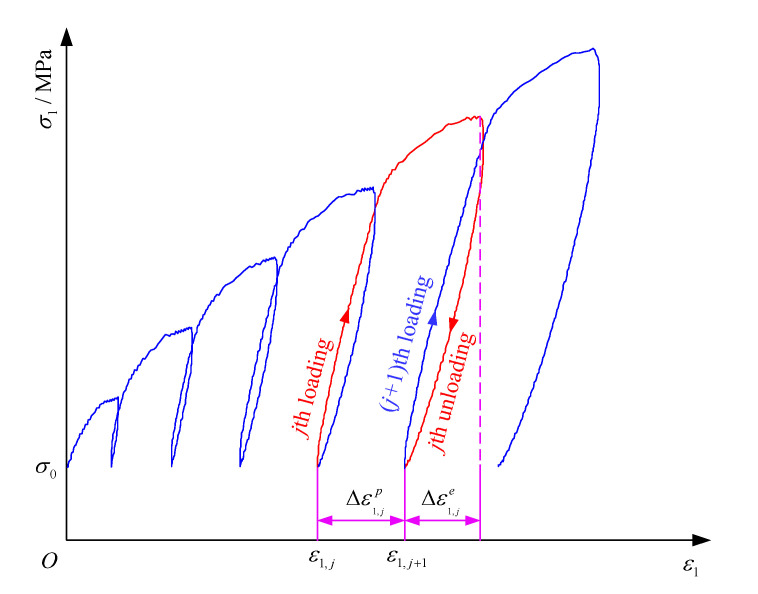
Schematic diagram of residual strain increment calculation.

Because the coal body has some defects such as pores and micro-fractures, after each loading and unloading, it cannot completely recover to the initial state, and residual deformation will occur on part of it, which is called plastic deformation. In order to quantitatively study the deformation characteristics during true triaxial cyclic loading and unloading, the residual strain increment and cumulative residual strain were used to describe the evolution of coal deformation parameters during true triaxial cyclic loading and unloading based on the stress–strain curves of coal cyclic loading and unloading. The calculation method of residual strain increment is illustrated in Figure 10. Therefore, the calculation expressions of residual strain increment and cumulative residual strain are as follows:

(1) $$\Delta \varepsilon_{i,j}^{p}=\varepsilon_{i,j+1}^{p}-\varepsilon_{i,j}^{p}$$

where $\Delta \varepsilon_{i,j}^{p}$ is the residual strain produced in the principal stress direction *i* during the *j*-th loading and unloading cycle, *i* = 1, 2, 3.

(2) $$\varepsilon_{i}^{p}=\sum_{j=1}^{N}\Delta \varepsilon_{i,j}^{p}$$

where $\varepsilon_{i}^{p}$ is the cumulative residual strain produced in the principal stress direction *i*, *i* = 1, 2, 3; *N* is the total number of cyclic loading and unloading.

**Figure 11:**
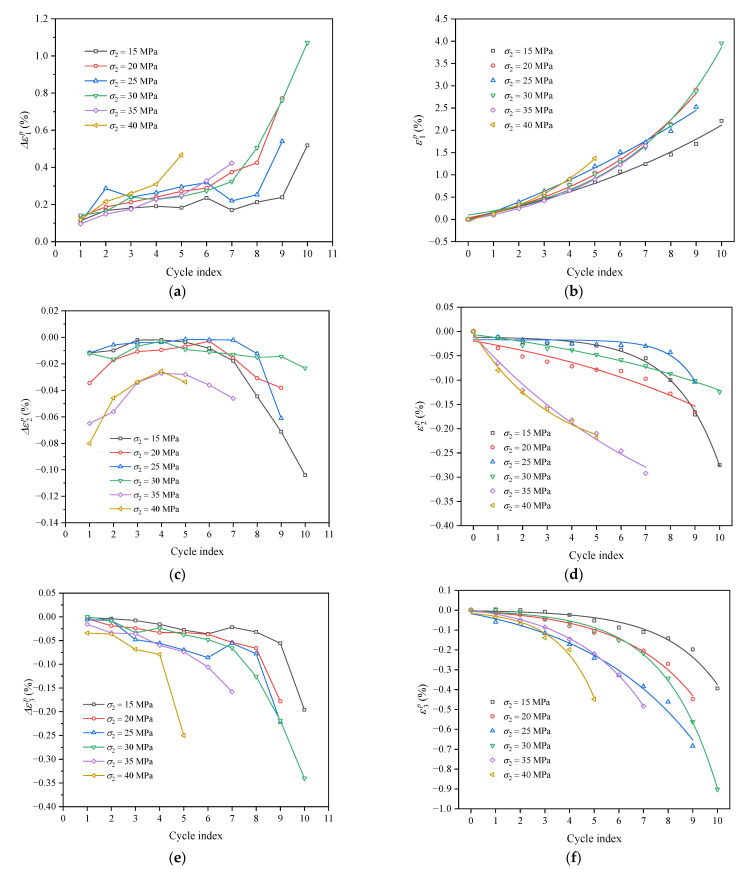
Evolution curves of residual strain increment and cumulative residual strain in the principal stress directions: (**a**) $\Delta \varepsilon_{1}^{p}$; (**b**) $\varepsilon_{1}^{p}$; (**c**) $\Delta \varepsilon_{2}^{p}$; (**d**) $\varepsilon_{2}^{p}$; (**e**) $\Delta \varepsilon_{3}^{p}$; (**f**) $\varepsilon_{3}^{p}$.

Residual strain increment and cumulative residual strain during each loading and unloading cycle can be calculated according to Equations (1) and (2). Additionally, the variation curves of residual strain increment and cumulative residual strain in the three principal stress directions with the increase in the number of cycles are displayed in Figure 11.

It can be observed from Figure 11a,c,e that the maximum residual principal strain increment ($\Delta \varepsilon_{1}^{p}$) and the minimum residual principal strain increment ($\Delta \varepsilon_{3}^{p}$) of coal increased relatively slowly at the initial stage of cycle. With the increase in the number of cycles, the damage to coal intensified until it gradually fractured. At this time, $\Delta \varepsilon_{1}^{p}$ and $\Delta \varepsilon_{3}^{p}$ began to surge. The increment of intermediate residual principal strain fell first and then rose with the increase in the number of cycles. The phenomenon was particularly evident when the intermediate principal stress was large ($\sigma_{2}$ = 35 MPa or 40 MPa), primarily due to the large difference between $\sigma_{2}$ and $\sigma_{3}$. At the initial stage of cyclic loading and unloading, the increase in $\Delta \varepsilon_{2}^{p}$ was restrained, and the lateral residual strain was transformed into an increase in $\Delta \varepsilon_{3}^{p}$. At the later stage of cyclic loading and unloading, the coal was gradually broken, and the lateral residual deformation began to transform into increases in $\Delta \varepsilon_{2}^{p}$ and $\Delta \varepsilon_{3}^{p}$. As observed in Figure 11b,d,f, the maximum cumulative residual principal strain ($\varepsilon_{1}^{p}$), the intermediate cumulative residual principal strain ($\varepsilon_{2}^{p}$) and the minimum cumulative residual principal strain ($\varepsilon_{3}^{p}$) all grew exponentially during cyclic loading and unloading. The axial residual strain ($\varepsilon_{1}^{p}$) produced in the cyclic loading and unloading process was distinctively larger than the lateral residual strain ($\varepsilon_{2}^{p}$ and $\varepsilon_{3}^{p}$), and $\varepsilon_{3}^{p}$ was larger than $\varepsilon_{2}^{p}$. This demonstrates that as cyclic loading proceeded continuously, compressive deformation in the direction of $\sigma_{1}$ and dilatancy deformation in the direction of $\sigma_{3}$ increased continuously, finally reaching the ultimate bearing capacity of coal and leading to the failure of coal.

#### 4.1.2. Analysis of Damage Evolution Based on Residual Strain

From the previous section, it was found that a certain amount of residual strain appeared during the cyclic loading and unloading. In order to quantitatively study the damage development of coal in the true triaxial cyclic loading and unloading deformation and failure process, this paper defines the damage variables according to the residual strain characteristics [29]:

(3) $$D_{i}=\frac{\sum_{j=1}^{n}\Delta \varepsilon_{i,j}^{p}}{\varepsilon_{i}^{p}}$$

where $D_{i}$ (0 ≤ $D_{i}$ ≤ 1 *i* = 1, 2, 3) are the damage variables in the directions of maximum principal stress, intermediate principal stress and minimum principal stress, respectively; $\Delta \varepsilon_{i,j}^{p}$ is the residual strain produced in the principal stress direction *i* during the *j*-th loading and unloading cycle; $\varepsilon_{i}^{p}$ is the cumulative residual strain produced in the principal stress direction *i*; *n* is the number of cyclic loading and unloading.

**Figure 12:**
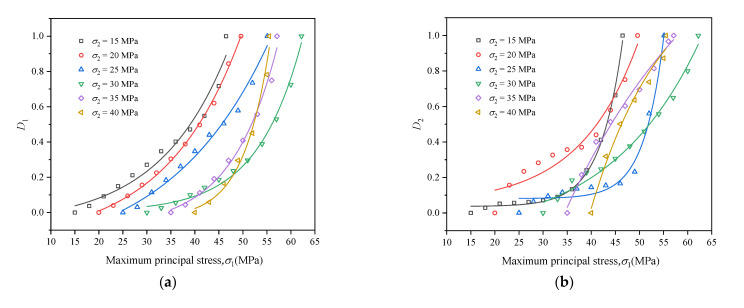

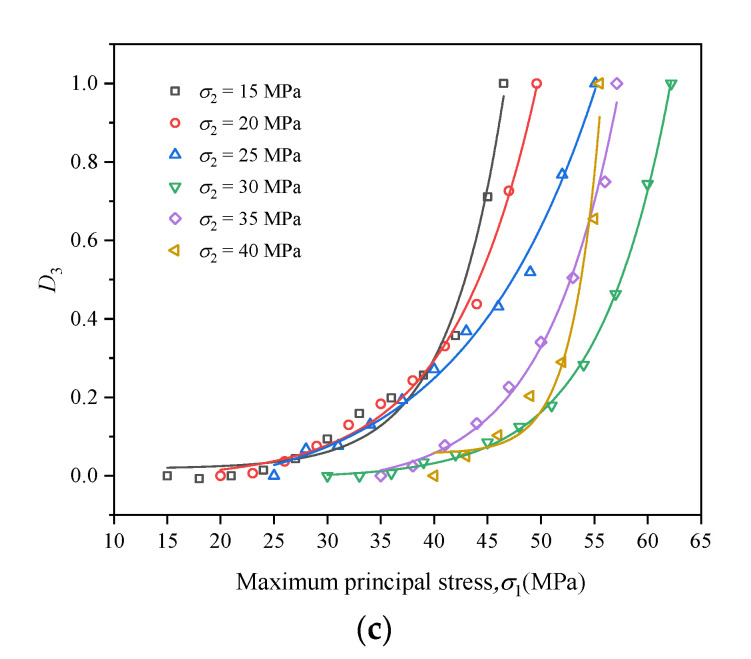
Curves of coal damage variable during triaxial cyclic loading and unloading: (**a**) $D_{1}$; (**b**) $D_{2}$; (**c**) $D_{3}$.

Through the damage variable calculation Equation (3), the coal damage variable $D_{i}$ in the three principal stress directions during true triaxial cyclic loading and unloading can be calculated, respectively. The calculation results are exhibited in Figure 12.

As shown in Figure 12, the variation trends of coal damage variables $D_{1}$ and $D_{3}$ under different intermediate principal stress were mostly similar. At the initial stage of cyclic loading and unloading, $D_{1}$ and $D_{3}$ increased slowly. As cyclic loading proceeded, they grew exponentially, and its damage variable equation can be expressed as $D_{i}=A_{0}+A_{1}e^{B_{1}\sigma_{1}}$. When $\sigma_{2}$ ≤ 30 MPa, it also conforms to the above damage variable equation. However, when $\sigma_{2}$ = 35 MPa or 40 MPa, the curve $D_{2}$-$\sigma_{1}$ shows an “upward convex” change, which means that the damage variable $D_{2}$ surges first and then rises slowly with the increase in cyclic load. Its damage variable equation can be expressed as $D_{2}=A_{2}\sigma_{1}^{2}+B_{2}\sigma_{1}+C(A_{2}\neq 0)$, which indicates that when $\sigma_{2}$ is large, it will cause a higher degree of damage to coal at the initial stage of cyclic loading and unloading, thus causing low strength of coal during cyclic loading and unloading. This also explains why $\sigma_{2}$ mainly exerts damage to coal and accelerates the destruction of coal when it is higher than 30 MPa.

### 4.2. Evolution Law of Energy Dissipation and Damage Characteristics

#### 4.2.1. Energy Distribution and Evolution Law

The residual strain produced during cyclic loading and unloading can only reflect the plastic deformation characteristics at the loading point and unloading point. In contrast, since an obvious hysteretic effect was produced in the stress–strain curve during cyclic loading and unloading, the area encircled by the loading and unloading curves can reflect the energy dissipation characteristics in each cyclic loading and unloading cycle [30] and, thus, describe the damage characteristics of coal during cyclic loading and unloading from the perspective of energy dissipation.

It was assumed that the test system was closed in which coal did not exchange heat with the outside world during true triaxial cyclic loading and unloading. According to the principle of energy conservation,

(4) $$U=U^{d}+U^{e}$$

where U is the total input energy density; $U^{d}$ is the dissipated energy density; $U^{e}$ is the elastic strain energy density.

**Figure 13:**
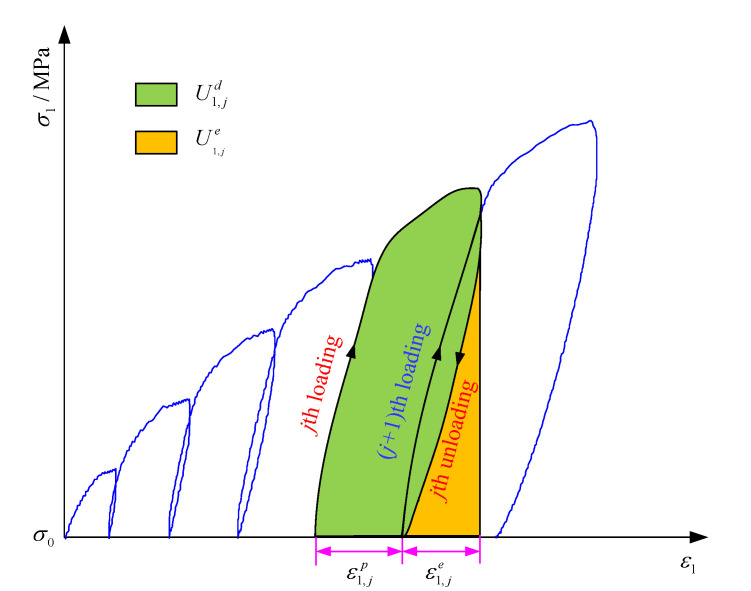
Schematic diagram of calculation of elastic strain energy density and dissipated energy density.

According to the stress–strain curve of coal during true triaxial cyclic loading and unloading, the densities of dissipated energy and elastic energy during loading and unloading can be calculated. As shown in Figure 13, the area under the *j*-th loading curve was the total energy density $U_{1,j}$ absorbed by the coal; the area under the *j*-th unloading curve was the elastic energy density $U_{1,j}^{e}$; and the area encircled by the *j*-th loading and unloading curves was the dissipated energy density $U_{1,j}^{d}$. On this basis, the calculation formulas of total energy density, elastic energy density and dissipated energy density during the *j*-th loading and unloading cycle can be obtained:

(5) $$U_{j}==\sum_{i=1}^{3}U_{i,j}=\sum_{i=1}^{3}\int \sigma_{i,j}d\varepsilon_{i,j}$$

(6) $$U_{j}^{e}==\sum_{i=1}^{3}U_{i,j}^{e}=\sum_{i=1}^{3}\int \sigma_{i,j}d\varepsilon_{i,j}^{e}$$

(7) $$U_{j}^{d}=U_{j}^{}-U_{j}^{e}$$

where $\sigma_{i,j}$ and $\varepsilon_{i,j}$ are the principal stress and strain of coal in the direction of *i* during the *j*-th loading and unloading, respectively; $U_{j}$, $U_{j}^{d}$ and $U_{j}^{e}$ are the total energy density, dissipated energy density and elastic energy density of coal during the *j*-th loading and unloading cycle, respectively.

**Figure 14:**
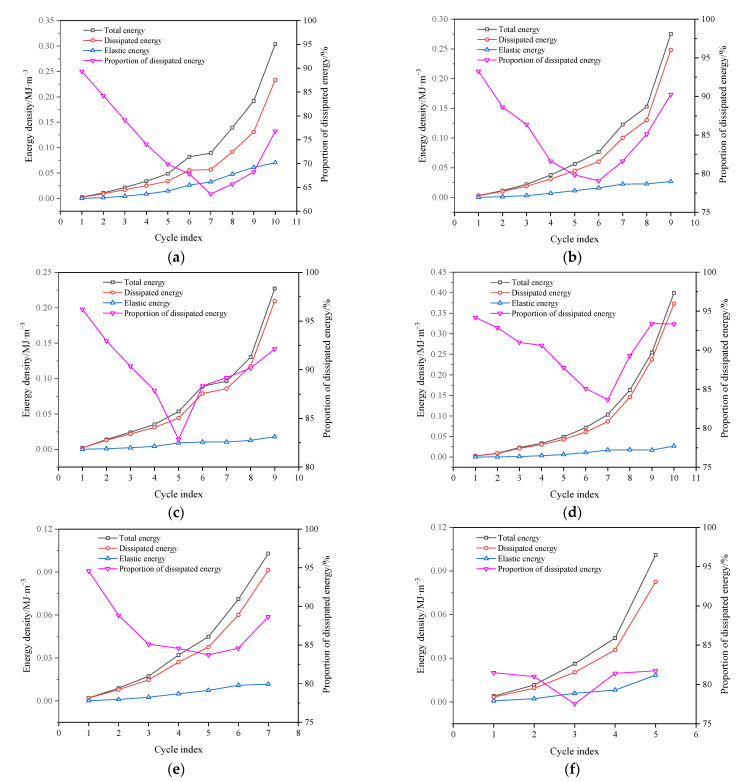
Distribution and evolution curves of coal energy densities during true triaxial cyclic loading and unloading: (**a**) $\sigma_{2}$ = 15 MPa; (**b**) $\sigma_{2}$ = 20 MPa; (**c**) $\sigma_{2}$ = 25 MPa; (**d**) $\sigma_{2}$ = 30 MPa; (**e**) $\sigma_{2}$ = 35 MPa; (**f**) $\sigma_{2}$ = 40 MPa.

The energy densities of coal in each cycle under different intermediate principal stresses can be calculated through Equations (5)–(7), based on which the distribution and evolution curves of coal energy densities during true triaxial cyclic loading and unloading can be obtained (Figure 14).

As can be seen from Figure 14, the energy distributions and evolutions of coal during cyclic loading and unloading under different intermediate principal stresses were similar on the whole. At the initial stage of cyclic loading and unloading, both elastic energy density and dissipated energy density increased slowly, but the proportion of dissipated energy density was much higher than that of elastic energy density. The primary reason for this was that the loose coal samples contained many primary fractures. Consequently, at the initial stage of loading and unloading, the primary fractures were gradually compacted, which consumed massive energy. As the number of cycles increased, the proportion of dissipated energy density gradually shrank, but most total input energy was still converted into the dissipated energy for plastic deformation. At the later stage of cyclic loading and unloading, the elastic energy density increased gradually in a small range. Meanwhile, the energy dissipation grew exponentially, and the proportion of energy dissipation density began to increase gradually. At this time, macroscopic fracture surface was produced in the coal body, which consumed much energy; additionally, the coal body gradually changed from a stable structure to a fractured structure, so that its bearing capacity deteriorated until it was entirely lost.

#### 4.2.2. Analysis of Damage Evolution Based on Energy Dissipation

Dissipated energy was mainly used for plastic deformation of coal during cyclic loading and unloading, and dissipated energy density can reflect the damage degree of coal. Therefore, the damage variable $D_{d}$ was defined as follows:

(8) $$D_{d}=\frac{\sum_{j=1}^{n}U_{j}^{d}}{U^{d}}$$

**Figure 15:**
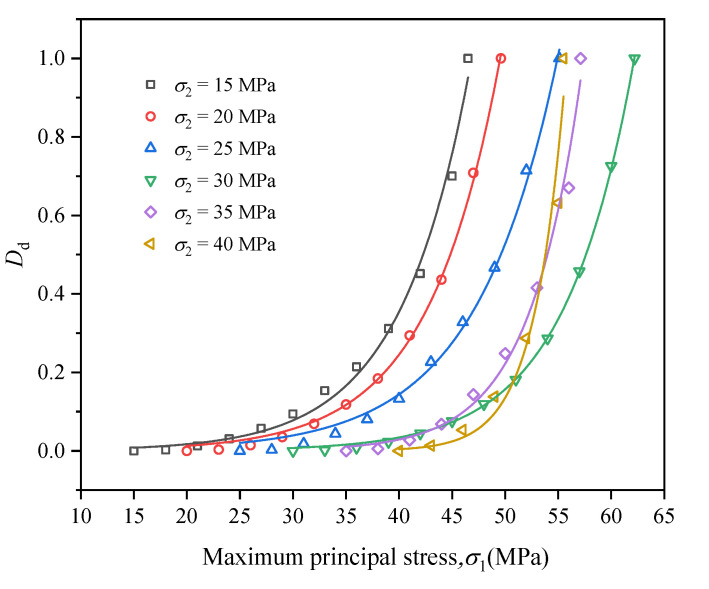
Evolution curves of coal damage variable during true triaxial cyclic loading and unloading.

Based on the energy evolution law of coal in the cyclic loading and unloading process, the damage variable $D_{d}$ of coal in the cyclic loading and unloading process under different intermediate principal stresses were fitted, and the fitting equation was as follows:

(9) $$D_{d}=A_{3}e^{B_{3}\sigma_{1}}$$

where $A_{3}$ and $B_{3}$ are both fitting parameters, and the evolution curves of coal damage variable during true triaxial cyclic loading and unloading are presented in Figure 15. The fitting parameters are listed in Table 1.

It can be seen from Figure 15 that the coal damage variables $D_{d}$ under different intermediate principal stresses all conformed to the damage variable equation $D_{d}=A_{3}e^{B_{3}\sigma_{1}}$, the fitting correlation coefficient *R*^2^ being above 0.96. The coal damage variable $D_{d}$ increased slowly at the initial stage of cyclic loading and unloading, and surged exponentially with the gradual increase in cyclic load. When $\sigma_{2}$ ≤ 30 MPa, with the increase in $\sigma_{2}$, the increasing speed of $D_{d}$ gradually decreased. At this time, $\sigma_{2}$ limited the fracturing-induced or destruction-induced energy dissipation of coal, which played a certain role in protecting coal and, thus, improved the energy storage limit of coal. When $\sigma_{2}$ > 30 MPa, with the increase in $\sigma_{2}$, the increasing speed of $D_{d}$ gradually increased. At this time, $\sigma_{2}$ caused damage to coal, which was not conducive to energy storage in coal. The above phenomenon explained the effect of intermediate principal stress in the true triaxial cyclic loading and unloading test from the perspective of energy dissipation.

## 5. Conclusions

In this paper, triaxial cyclic loading and unloading tests were performed on coal under different $\sigma_{2}$ values. On this basis, the full stress-strain curves of coal samples were obtained, and the evolution laws of strength, fracture characteristics, deformation parameters and energy of coal under different conditions were discussed. Furthermore, the evolution characteristics of coal damage variable during true triaxial cyclic loading and unloading were quantitatively analyzed from the perspectives of deformation and energy dissipation. The main conclusions are as follows:

1. The envelope shape of stress–strain curve of coal in the true triaxial cyclic loading and unloading test resembled that in the true triaxial loading test. In the true triaxial cyclic loading and unloading test, an obvious hysteretic effect was observed in the stress-strain curve. In addition, the area of the hysteretic loop grew gradually with the increase in the number of cycles, reaching the maximum near the peak strength of coal body. Finally, the plastic flow failure of coal body occurred. With the increase in $\sigma_{2}$, the strength of coal body presented a change trend of increasing first and then decreasing, and the intermediate principal stress gradually changed from protecting the coal body to damaging it.
2. The failure modes of coal under the two loading paths were both shear failure and tensile failure caused by axial compression. During true triaxial loading, the sample experienced Y-shaped failure and had few secondary fractures. In contrast, during true triaxial cyclic loading and unloading, the failure was approximately X-shaped and accompanied by numerous secondary fractures and debris. With the increase in $\sigma_{2}$, the failure mode gradually changed from shear failure to tensile failure.
3. In the true triaxial cyclic loading and unloading test, the residual strain of coal increased exponentially with the number of cycles, and the damage variable was defined based on the residual strain characteristics of coal. When $\sigma_{2}$ ≤ 30 MPa, the coal damage variables $D_{1}$, $D_{2}$ and $D_{3}$ increased exponentially with the increase in cyclic load. When $\sigma_{2}$ > 30 MPa, the damage variable $D_{2}$ soared first and then rose slowly with the increase in cyclic load.
4. The distributions and evolutions of coal energy under different intermediate principal stresses were mostly similar. With the increase in the number of cycles, the dissipated energy density increased exponentially, and the dissipated energy ratio decreased first and then increased. Damage variable based on energy dissipation can describe damage evolution during cyclic loading and unloading. Under different intermediate principal stresses, the coal damage variables $D_{d}$ all conformed to the equation $D_{d}=A_{3}e^{B_{3}\sigma_{1}}$.

## Figures and Tables

**Table 1:** Fitting parameters.

$\sigma_{2}/\mathrm{MPa}$	*A*_3_/10^−4^	*B*_3_	*R* ^2^
15	8.17183	0.15182	0.99235
20	6.68289	0.14762	0.99856
25	8.00289	0.12981	0.99568
30	0.775896	0.1522	0.99964
35	8.31499 × 10^−2^	0.20385	0.98773
40	4.35293 × 10^−5^	0.34505	0.96714

## Data Availability

The data used to support the findings of this study are included within the article.
